# MR-guided focused ultrasound for the novel and innovative management of osteoarthritic knee pain

**DOI:** 10.1186/1471-2474-14-267

**Published:** 2013-09-13

**Authors:** Masashi Izumi, Masahiko Ikeuchi, Motohiro Kawasaki, Takahiro Ushida, Kazuo Morio, Hirofumi Namba, Thomas Graven-Nielsen, Yasuhiro Ogawa, Toshikazu Tani

**Affiliations:** 1 Department of Orthopaedic Surgery, Kochi Medical School, Kochi University, Oko-cho Kohasu, Nankoku 783-850, Japan; 2 Multidisciplinary Pain Center, School of medicine, Aichi Medical University, Nagakute, Japan; 3 Department of Radiology, Kochi Medical School, Kochi University, Nankoku, Japan; 4 Laboratory for Musculoskeletal Pain and Motor Control, Center for Sensory-Motor Interaction (SMI), Department of Health Science and Technology, Faculty of Medicine, Aalborg University, Aalborg, Denmark

**Keywords:** MR-guided Focused Ultrasound (MRgFUS), Knee, Osteoarthritis, Pain

## Abstract

**Background:**

Severe knee pain associated with osteoarthritis (OA) is one of the most common and troublesome symptoms in the elderly. Recently, local bone denervation by MR-guided focused ultrasound (MRgFUS) has been demonstrated as a promising tool for pain palliation of bone metastases. The purpose of this study was to develop a novel treatment for knee OA using MRgFUS, and to validate its safety and efficacy.

**Methods:**

Eight patients with medial knee pain and eligible for total knee arthroplasty were included. MR-guided focused sonication treatments were applied to bone surface just below the rim osteophyte of medial tibia plateau with real-time monitoring of the temperature in the target sites. The pain intensity during walking was assessed on a 100 mm visual analog scale (VAS) before and after treatment. Pressure pain thresholds (PPTs) were also evaluated over several test sites adjacent to the sonication area and control sites one month after treatment.

**Results:**

Six patients (75%) showed immediate pain alleviation after treatment, and four of them demonstrated long-lasting effect at 6-month follow up (mean VAS reduction; 72.6%). In responders, PPTs in medial knee were significantly increased after treatment (Median; pre- 358 kpa vs post- 534 kpa, p?<?0.0001). There were no adverse side effects or complications during and after treatment.

**Conclusions:**

These initial results illustrate the safety and efficacy of the newly developing MRgFUS treatment. Significant increase of PPTs on treated area showed successful denervation effect on the nociceptive nerve terminals. MRgFUS is a promising and innovative procedure for noninvasive pain management of knee OA.

**Trial registration:**

Trial Registration: UMIN000010193

## Background

Knee Osteoarthritis (OA) ranks among the most common disabling arthritic conditions in the elderly [1]. A major symptom of knee OA is chronic knee pain which has a significant effect on patients’ quality of life [2]. There are several conservative options for pain management, including physical therapy, use of non-steroidal anti-inflammatory drugs, intraarticular injection with steroids or hyaluronic acids [3]. However, these treatments are not sufficient to control severe knee OA pain [4]. Although total knee arthroplasty (TKA) is a validated and reliable intervention for alleviating severe knee pain [5], there are some patients who are at high risk during surgery and other patients who are not willing to undergo surgery. The number of these patients is expected to increase because of population aging, therefore, it is necessary to explore additional nonsurgical treatments for knee OA to achieve better pain relief.

MR-guided focused ultrasound (MRgFUS) treatment is a noninvasive technique that enables to perform localized thermal ablation by focusing the acoustic energy precisely to the targeted sites [6]. Three-dimensional treatment planning and continuous real-time monitoring of the temperature in the target sites by MR imaging are the two crucial advantages of this system [7]. Clinically, the feasibility and effectiveness of MRgFUS have been evaluated in several benign and malignant tumors such as uterine fibroids [8,9], breast cancer [10,11], and brain tumors [12]. In recent years, palliative therapy of bone pain due to metastasis has been recognized as a promising alternative treatment with the mechanism of local bone denervation [13-15]. As for a treatment of chronic musculoskeletal pain, however, there was only one case-series of MRgFUS application to osteoarthritic lumbar facet joints, which targeted periosteum around facet joint to achieve local bone denervation and reported safe and effective outcomes against low back pain [16]. In knee OA, tenderness of the bony margins of the joint is a quite common symptom involved in American College of Rheumatology criteria for clinical diagnosis [17], which might be caused by rich nociceptive nerve terminals in this area. Therefore, it is reasonable to assume that the same mechanism as facet joint treatment is certainly available to alleviate joint pain caused by knee OA. The purpose of this study was to develop a novel treatment for knee OA using MRgFUS, and to validate its safety and efficacy in an initial case series.

## Methods

### Patients

This case series study was carried out with the approval of the Institutional Review Board and in a prospective, non-controlled manner. All patients were informed about the intervention prior to treatment, and written consent for participation and publication of individual clinical details were obtained. Participation was voluntary and did not preclude other treatment options. The study and all interventions were carried out in the Department of Orthopaedic Surgery in Kochi university hospital between December 2010 and April 2012. Patients complaining severe medial knee pain associated with radiological OA were recruited for this study. Inclusion criteria were age older than 60 years, previous conservative treatments longer than 3 months, and pain scores on an visual analog scale (VAS, 100 mm) greater than 40 mm during walking. Radiological inclusion was restricted to grade 4 medial knee OA according to Kellgren-Lawrence classification [18], because the patients could be salvaged by TKA conversion. Exclusion criteria were contraindications for MRI, psychiatric conditions, and allergies of local anesthetics.

In this series, eight patients (6 female, 2 male) with the mean age of 78 (± 6.4; standard deviation) years were treated. The mean clinical score (Japanese Orthopedic Association score for knee OA) was 48 (± 5.3) points (maximum 100 points: domains are pain on walking or stair stepping, range of motion, and joint effusion). All patients were eligible for TKA, and half of them were scheduled surgery and underwent MRgFUS treatment during waiting period. The others were scheduled only for MRgFUS treatment because they were not willing to undergo surgery.

### MR-guided focused ultrasound procedures

**Figure 1:**
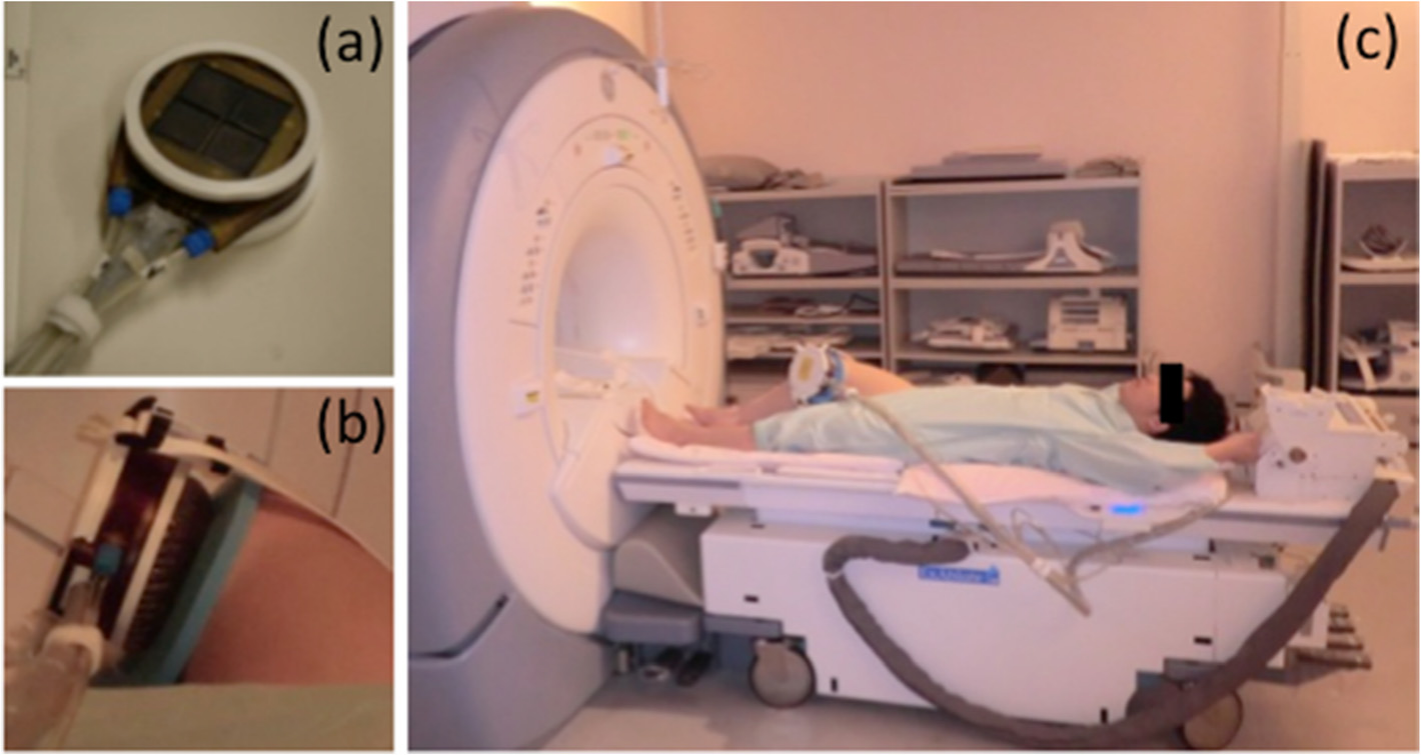
**System set up. (a)** Conformal sonication devise **(b)** Strapped onto the medial knee. Chilled water is circulated within a semi-permeable membrane. **(c)** Full view of the patient.

**Figure 2:**
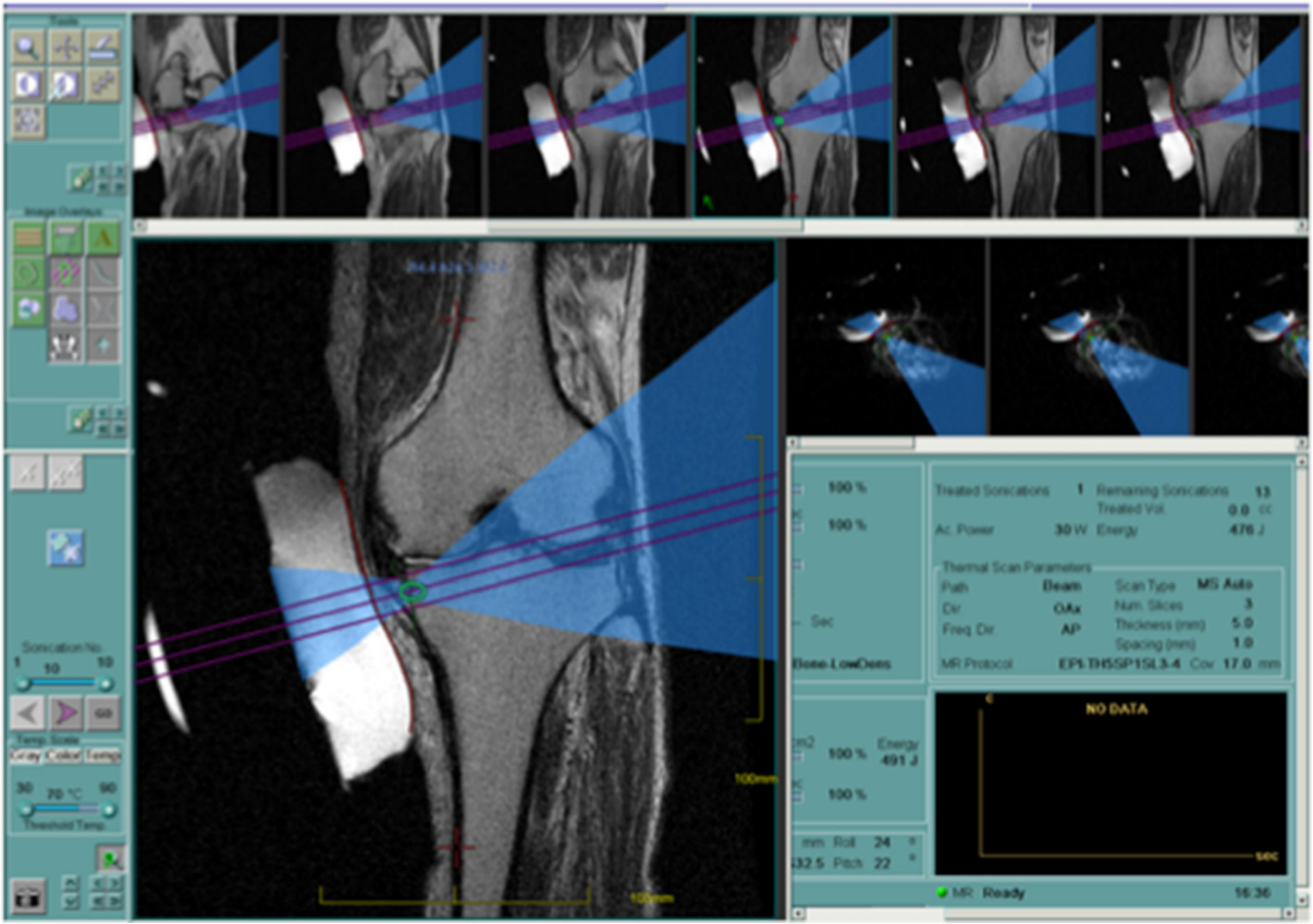
**Three-dimensional treatment planning.** Sonication site and ultrasound beam pathway are indicated by the examiner. The system automatically generated the optimal treatment plan including energy levels and number of sonications.

**Figure 3:**
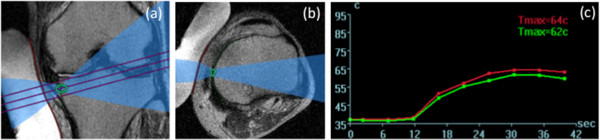
**Real-time monitoring.** Current sonication spot (green circle) and ultrasound beam pathway are shown in Coronal view **(a)** and axial view **(b)**. Temperature elevation is monitored by two curves indicated mean (green) and maximum (red) temperature in the spot **(c)**.

The treatment was conducted as an outpatient setting, using the MRgFUS system (ExAblate® 2100, InSightec Ltd, Haifa, Israel) integrated with an MRI scanner (GE Signa EXCITE 3.0 T MRI, Milwaukee, WI, USA). In this series, the criteria of sonication area was determined as the bone surface just below the rim osteophyte of medial tibia plateau, which is the insertion site of deep medial collateral ligament. Patients underwent local anesthesia with 15 ml of 0.75% ropivacaine around the periosteum in treatment sites and lay supine on the MRI table. A conformal sonication device was strapped onto the medial knee (Figure 1). This is a newly developed transducer and chilled water is circulated within a semi-permeable membrane to provide acoustic coupling and cool the skin during treatment. Coronal, sagittal, and axial unenhanced T2-weighted MR images were obtained and loaded into MRgFUS workstation to allow accurate three-dimensional planning and targeting of the lesion. The outline of bone surface as well as skin and the area to be treated were carefully drawn on the planning images of coronal and axial view. The system automatically generated the optimal treatment plan including energy levels and number of sonications (Figure 2). The ultrasound beam was angled to avoid popliteal neurovascular bundles. Initially, a low energy test sonication was performed to ensure safety and accuracy of the procedure. Then, therapeutic sonications began with higher energy to achieve ablation. Throughout the treatment, the location of each sonication and the temperature elevation in the tissue adjacent to the target area were monitored in real time (Figure 3). The temperature elevation was aimed at 60°C, and treatment parameters such as energy, sonication duration or spot size were modified in response to the monitoring. The patients held a stop switch and were able to interrupt anytime during the treatment.

### Outcome measures

The primary outcome measure was VAS scores during walking on a scale graduated from 0 (no pain) to 100 mm (maximal pain). A response to treatment was defined as a 50% or greater decrease in the pain VAS according to a proposal of Outcome Measures in Rheumatology Clinical Trials and Osteoarthritis Research Society International [19]. The VAS was collected before, 3 days, 10 days, and 1 month after the MRgFUS in all patients and in some patients additional VAS scores were obtained at 3, 6, 12, and 18 months.

In addition, pressure pain thresholds (PPTs) were measured over 6 test sites adjacent to the sonication area and at 2 control sites. The test sites in medial knee were; A: anterior joint space; B: middle joint space; C: posterior joint space; D: anterior tibia plateau; E: middle tibia plateau; F: posterior tibia plateau. All sites were easily identified based on the location of joint space, medial collateral ligament and tibial osteophyte. As control sites, lateral joint space and ipsilateral upper arm (3 cm proximal to the humerus insertion of deltoid muscle) were examined. A handheld algometer (Commander, J Tech Medical Industries, Heber city, UT, USA) with a 1 cm^2^ probe was used to record PPTs. The PPT was defined as the first point at which patients perceived the pressure as slight pain. PPTs were measured by single examiner (MI) at pre- and one month post-treatment. Prior to the pre-treatment assessment, high intra-rater reliability was confirmed in each patient. PPTs were recorded two times on each site and the mean threshold was used for statistical analysis.

Radiological assessments were performed at 3 days, 1 month, 6 months and 12 months by X-ray, at 3 months by routine plain MRI of the knee. The treated bone sample was taken from patients who underwent TKA after MRgFUS treatment and histopathological evaluation was performed using hematoxylin and eosin staining.

### Statistical analysis

The PPT data are presented as median and interquartile range in text and figures. Kruskal-Wallis test followed by Dunn’s test was used to compare PPTs among eight sites at pre-treatment. Wilcoxon signed rank test was used for comparison of difference between pre- and post-treatment PPTs in medial knee (including 6 sites; A-F) and on each site. Significant difference was set at *p*?<?0.05.

## Results

The mean time used for preparing the system was 86 minutes (50–120 min) while the mean treatment time was 74 minutes (50–120 min). The mean therapeutic energy level was 735 Joules (491–952) and the mean number of sonication was 12.4 (10–20) per patient. The mean follow-up period was 9 (6–18) months after treatment. There were no adverse side effects or complications reported during and after treatment.

### Pain intensity effects

**Figure 4:**
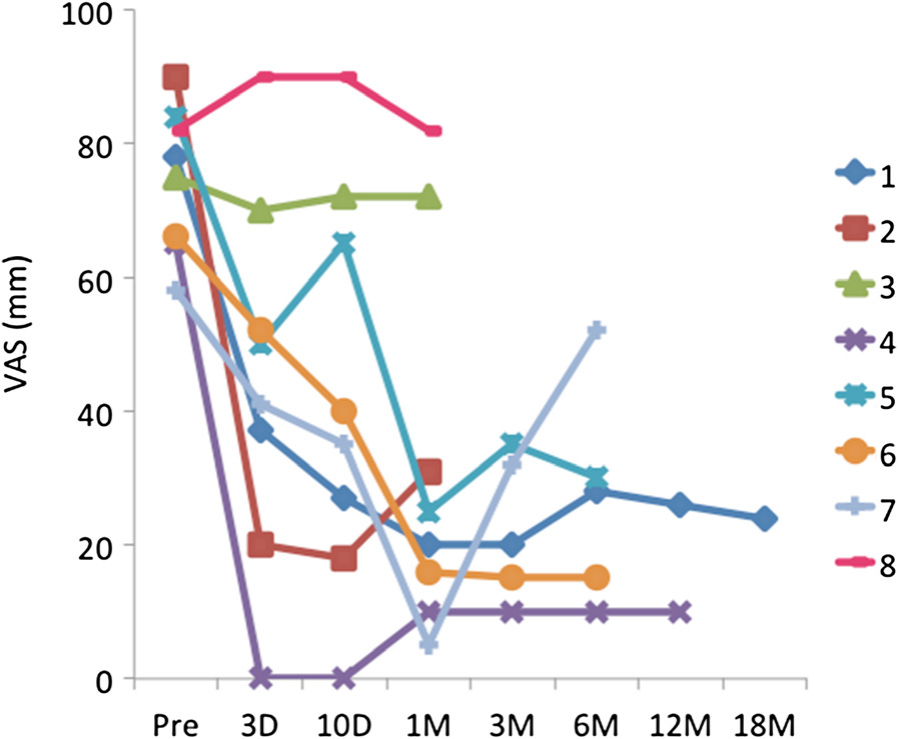
**VAS scores of the pain intensity during walking at each follow-up time point.** Six patients (Case 1, 2, 4, 5, 6 and 7) showed good pain relief after treatment.

The VAS scores were reduced 3 days, 10 days, and one month compared with pre-treatment in the 6 responders (Figure 4). In particular, four patients (Case 1, 4, 5, and 6) had long-lasting pain alleviation (mean VAS reduction at 6 months: 72.6%). One patient (Case 7) showed recurrence of pain at 6-month follow up. Two patients (Case 2 and 3) underwent total knee arthroplasty one month after MRgFUS treatment. One of the non-responders (Case 8) dropped out and switched to opioid therapy one month after MRgFUS treatment.

### Pressure pain sensitivity

**Figure 5:**
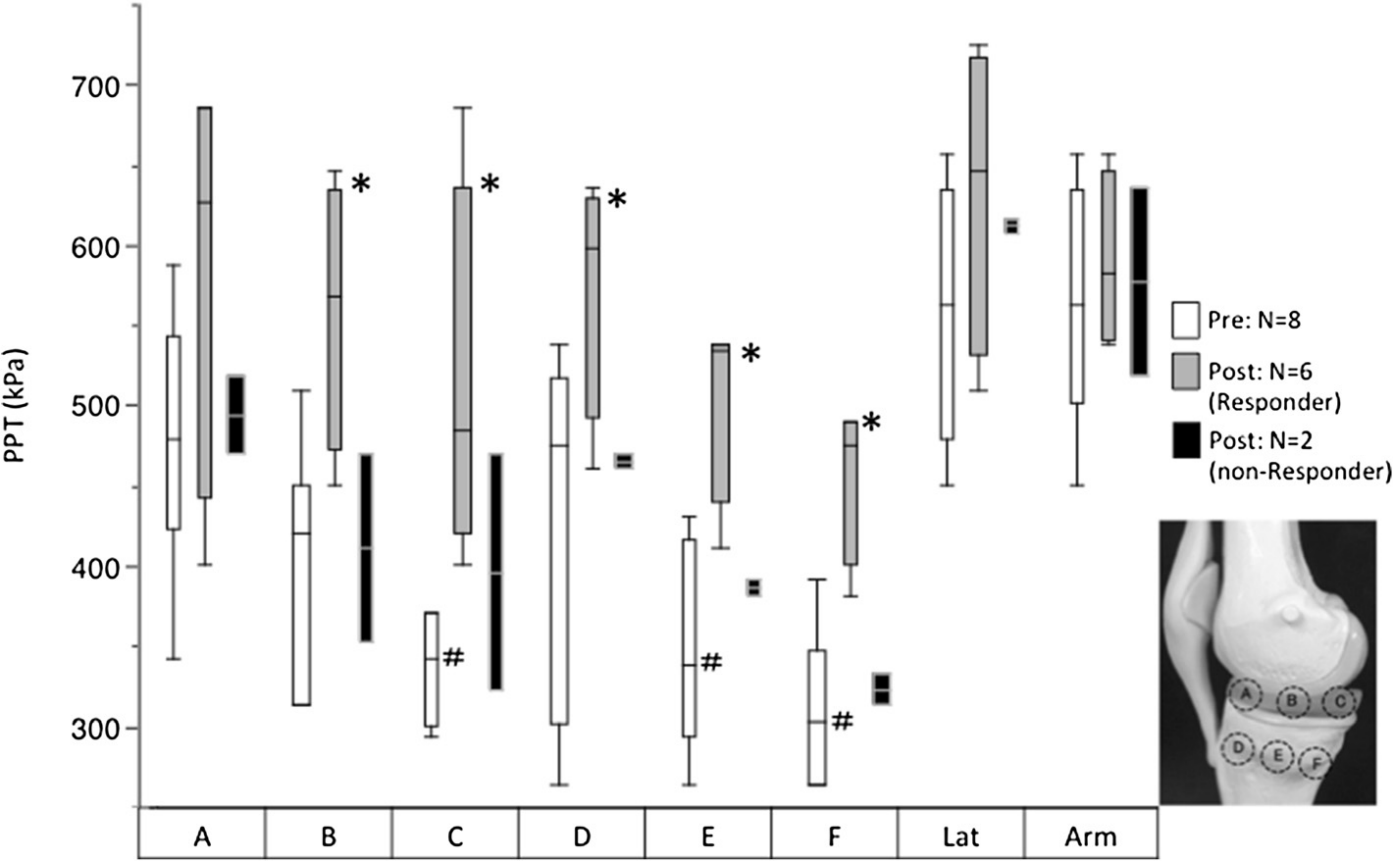
**PPTs at pre- and post-treatment.** White bar indicates pre- treatment PPTs (n?=?8). Gray and black bar indicate post-treatment PPTs of responder (n?=?6) and non-responder (n?=?2), respectively. # : *p*?<?0.05 compared with Arm at pre-treatment. *: *p*?<?0.05 compared with pre-treatment in responders. Sonications were applied to the site D, E, F.

At pre-treatment and compared with the arm, the middle and posterior tibia plateau as well as posterior joint space showed significant lower PPTs (P?<?0.05; Figure 5). In the 6 responders, the PPTs in medial knee were 358 kpa [290 - 431]at pre-treatment and 534 kpa [461 - 605]at post-treatment, which showed significant difference (p?<?0.0001). In site-specific evaluation, the PPTs on middle, posterior joint space and tibia plateau were significantly increased after treatment (P?<?0.05; Figure 5), suggesting that the nociceptive nerve terminals in the medial knee were successfully treated. In the two non-responders, the PPT values post-treatment were comparable with the pre-treatment values.

### Histopahological evaluation

**Figure 6:**
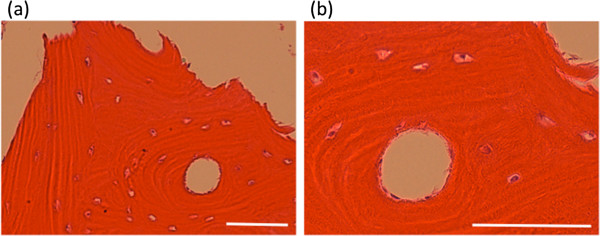
**Histology of the bone sample in the treated area. (a)** low magnification, **(b)** high magnification. No significant focal bone necrosis was observed. Scale bar: 50 μm.

The cortical bone sample of the treated area was taken from 2 patients during TKA. Light microscopic assessment showed maintained bone morphology (Figure 6a) and normal osteocytes (Figure 6b), which demonstrated no significant focal bone necrosis due to MRgFUS treatment.

### Case

**Figure 7:**
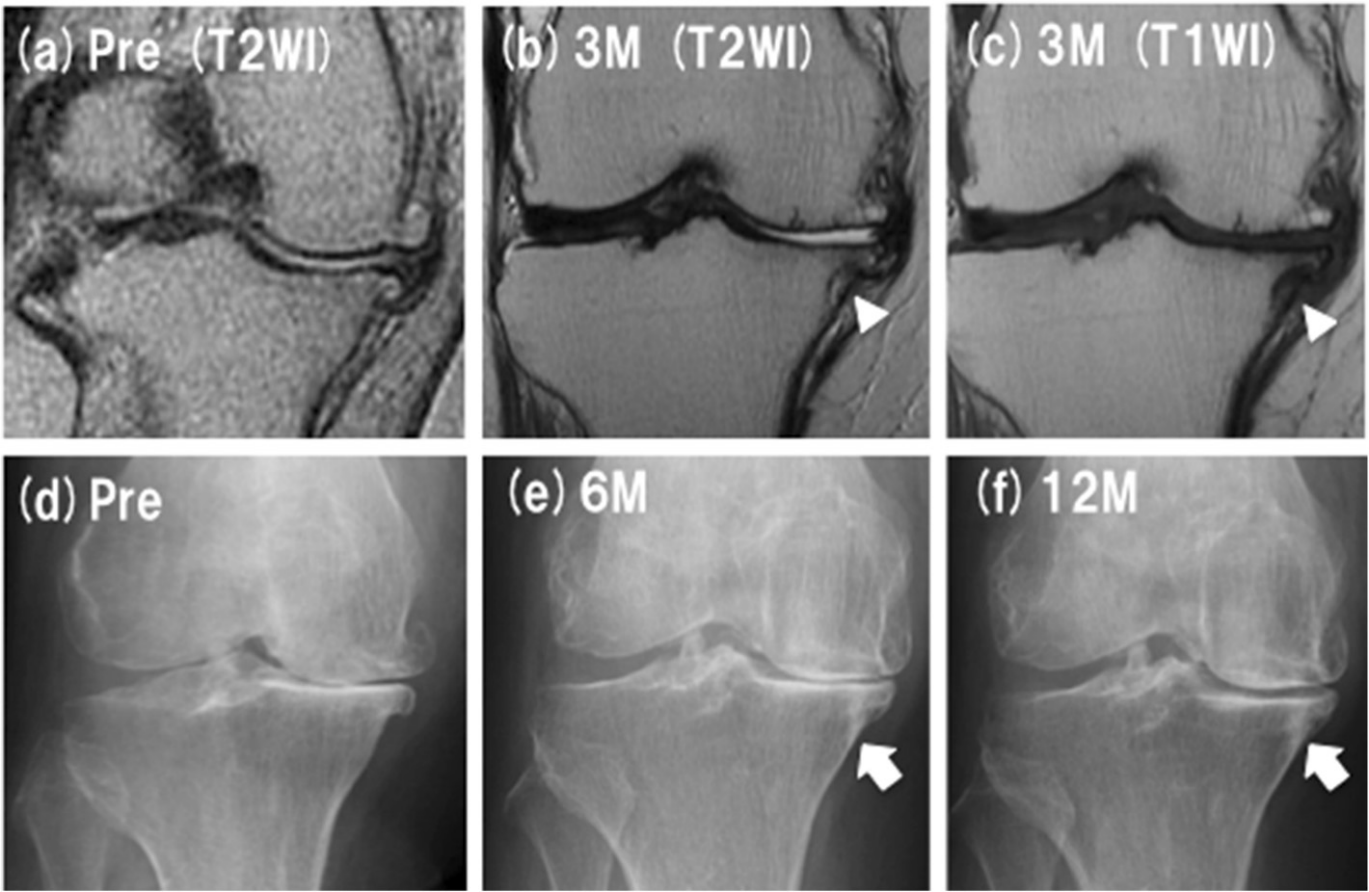
**Radiological changes between treatments. (a)** MRI (pre-, T2WI), **(b)** MRI (3 months post-, T2WI), **(c)** MRI (3 months post-, T1WI), **(d)** X-p (pre-), **(e)** X-p (6 months post-), **(f)** X-p (12 months post-). Arrow heads in MRI indicate low intensity curved line at the sonication site. Arrows in X-p indicate osteosclerotic change in accordance with the line in MRI.

A-82 year-old woman (Case 1) underwent MRgFUS treatment for her left knee. She had successfully undergone TKA for her right knee 5 months ago, and been scheduled surgery for her left knee. She had medial knee pain with the VAS of 78 mm and tenderness on her medial joint space and tibia plateau (pre-treatment median PPT values in the medial knee: 280 kPa). After treatment, she reported dramatic and long-lasting reduction in her left knee pain with PPTs increase (post-treatment median PPT values: 456 kPa). The clinical score was improved from 50 points in pre- to 75 points in post-treatment. At the 18-month follow-up, she was no longer suffering from severe knee pain in her daily life, and canceled her surgery. Figure 7 showed the radiological changes in this patient. MRI showed a low intensity curved line at the sonication site in T1 and T2 weighted images. In X-ray films, an osteosclerotic change was seen in accordance with the low intensity curved line in MRI. There were no findings of OA progression, osteonecrotic change, or segmental collapse of tibia plateau during follow-up period. The other patients also showed similar courses of radiographic change after treatment.

## Discussion

This is the first report of clinical application of MRgFUS for knee OA. Real-time monitoring of the sonication area and temperature elevation enabled performance of safe and accurate treatment. Even though the inclusion was restricted to most severe OA in this pilot study, 75% of patients showed successful pain relief. Similar to previous reports of bone metastases [13-15] or lumbar facet OA [16], the pain alleviation was rapid and long-lasting. Unlike a conventional transducer integrated with MRI table, a newly developed conformal sonication device was a good fit for extremities and enabled easier treatment of knee OA. Intravenous sedation and opioid administration were not necessary for our treatment, which were applied in the previous series of bone metastases [13-15] or low back pain [16]. Local anesthesia with ropivacaine around the periosteum was enough to reduce pain associated with sonication. Patients were able to relax throughout the procedure and to walk soon after treatment.

The mechanism of pain alleviation is most likely local denervation caused by the heat denaturation of the treated area. However, no previous studies have suggested an assessment method to estimate the denervated area of MRgFUS. In this regard, pressure algometry is a quite simple and useful tool for quantitative evaluation after denervation treatment. Reliable repeated PPT measurements around knee joint have been documented by means of locating the assessment sites in relation to bone landmark [20]. In the present study, all sites in medial knee were easily identified based on the location of joint space, medial collateral ligament and tibial osteophyte, enabling to retest PPTs in a reproducible manner. In our patients who responded to the treatment with pain reduction, PPTs on sonication area were significantly increased after treatment, which means that patients felt less pain by pressure stimulation after denervation. The patients who did not respond to the treatment did not show increased pressure pain thresholds which may suggest that an PPT increase would be a necessary condition of successful treatment. Future studies will be needed to verify if earlier follow-up assessments may be used to predict treatment success.

In this series, all sonications were applied to bone surface just below the rim osteophyte of medial tibia plateau. From a pathophysiological perspective it has been reported that sensory nerve invasion containing substance P and calcitonin gene related peptide was seen in tibial osteophyte in human OA patients [21]. Because surface area of the tibial rim osteophyte itself was a bit narrow to plan sonication, the base of the osteophyte was treated instead. Furthermore, lower PPTs were observed in this area at pre-treatment in all patients and this is also a general finding in OA knees [17]. In other words, hypersensitivity of nociceptive nerve terminals against pressure stimulation was seen in this area, which was preferable for denervation treatment. From a practical perspective there were other reasons to select the treated area. Firstly, bone is a better indication for MRgFUS than soft tissues. Lower thermal conductivity and higher ultrasound absorption rate of cortical bone allows the denervation treatment safe and efficient, which had been demonstrated in previous reports [13-16]. Secondly, tibial rim osteophyte was a good landmark for reproducible planning, treatment and assessment.

The osteosclerotic change after the treatment was interesting. In our patients, the temperature elevation of bone surface was aimed at 60°C because protein denaturation occurred above the temperature of 57–60°C for a few seconds [22,23]. The goal temperature was almost same as previous publications, and some authors found similar new bone formation after the treatment of bone metastases [13,14]. The mechanism of osteosclerotic change in the treated area is unknown. Although it cannot be excluded that minor thermal or non-thermal bone damage occurred, new bone formation might be an encouraging radiological finding of this therapy [24]. Including its relation to the long-lasting pain relief, further basic research of treated bone marrow would be necessary to assess this phenomenon.

According to the pain alleviation mechanism and results of this study, a good candidate for MRgFUS treatment is patient presenting with localized medial pain, lower PPTs around tibial osteophyte, and no bone marrow lesion or osteonecrosis. In this series, two patients did not respond the treatment. One patient complained spreading medial knee pain and the other had small bone marrow lesion in medial femoral condyle and tibia plateau. Detailed assessment of pain distribution, pressure pain sensitivity, and MRI examination before the treatment might be essential to achieve satisfactory results.

Percutaneous radiofrequency treatment has been reported as a beneficial local denervation therapy for knee OA [4,25]. Comparing with radiofrequency, MRgFUS treatment has some advantages. Closed-loop, real-time spatial and thermal monitoring enables the treatment safer and more accurate. Identifying target nerve is not trivial and the outcome is highly technique-dependent in radiofrequency [25]. MRgFUS treatment is not a technique-dependent procedure and low inter-operator variability is expected. MRgFUS treatment does not cause widespread hypoesthesia which often observed in radiofrequency treatment [25], because MRgFUS treats most peripheral zone of the sensory nerve. On the other hand, there are some obvious disadvantages of MRgFUS. Enormous initial cost of the treatment is most critical. In addition, patients of contraindications for MRI cannot undergo the treatment. Required time for the set up and treatment is also longer than radiofrequency.

This study has some limitations. First, the most important weakness is that it was a case series including small number of patients without control group. Hence, it is difficult to be sure that there were no placebo effects. However, 75% of patients showed successful pain relief along with significant increase of PPTs. Although further study with blinded and randomized controlled trial is required for constructing evidence, our initial results suggested the safety and efficacy of the treatment. Second, the inclusion was restricted most severe medial knee OA because this is a pilot study so that the patients should be salvaged by TKA conversion. Based on this study and the mechanism of pain relief, medial OA in earlier stage or lateral OA might possibly become a candidate for the treatment. Third, this study did not have a long follow-up period. Two patients underwent total knee arthroplasty and one non-responder dropped out one month after the treatment. Long-term effectiveness of MRgFUS treatment including ADL and QOL assessment should also be carried out in a continuing study.

## Conclusion

MRgFUS treatment had a potential of rapid and long-lasting pain alleviation without adverse side effects. Significant increase of PPTs on treated area showed successful denervation effect on the nociceptive nerve terminals. MRgFUS is a promising and innovative procedure for noninvasive pain management of knee OA.

## Competing interests

The authors have no competing interests to declare in regard to this manuscript.

## Authors’ contributions

MI was involved in the conception, planning and designing this study, the acquisition of data, analysis and interpretation of data, and writing the manuscript. MI, MK, TU and TGN were involved in planning and designing this study, analysis and interpretation of data, and critical revision of the manuscript for important intellectual content. KM and HN participated in the acquisition of data. YO and TT was involved in planning this study and drafting the manuscript. All authors read and approved the final manuscript.

## Pre-publication history

The pre-publication history for this paper can be accessed here:

http://www.biomedcentral.com/1471-2474/14/267/prepub
